# Disparities in utilization of sexual and reproductive health services among high school adolescents from youth friendly service implemented and non-implemented areas of Southern Ethiopia

**DOI:** 10.1186/s13690-020-00508-w

**Published:** 2020-12-01

**Authors:** Bayu Haile, Mulugeta Shegaze, Tesfaye Feleke, Mustefa Glagn, Eshetu Andarge

**Affiliations:** 1 Department of Emergency Outpatient, Gazer Primary Hospital, South Omo Zone, Southern Ethiopia Ethiopia; 2 School of Public Health, College of Medicine and Health Sciences, Arba Minch University, P.O.Box:021, Arba Minch, Southern Ethiopia Ethiopia

**Keywords:** Ethiopia, Adolescent, Utilization, Sexual and reproductive health, Youth friendly services

## Abstract

**Background:**

In recent years, much effort was made to improve access to sexual and reproductive health services (SRH) to adolescents and youths in Ethiopia particularly through establishment of youth friendly service (YFS) corners as part of the existing health care facilities. The existing evidences focused on investigating the utilization of SRH services at YFS established areas alone. There is a dearth of evidence which compares the SRH service use between the YFS implemented and non-implemented areas so that evidences can be drawn to suggest on the successes of the expansion of youth friendly corners.

**Methods:**

A school-based comparative cross-sectional study was conducted by employing a multistage cluster sampling method. A pre-tested self-administered questionnaire was used to collect data and the collected data were entered in to Epidata version 4.4.1 software and then exported to SPSS version 20 for analysis. χ^2^ test was used to see a significant difference in SRH service utilization among adolescents from YFS implemented and non-implanted areas. The association between the SRH services utilization and the independent variables were examined using binary logistic regression. Finally, variables having *p*-value less than or equal to 0.05 in the multivariable logistic regression model were considered as statistically significant.

**Results:**

There were a significant difference in the rate of SRH service utilization between YFS implemented (33.8%) and YFS non- implemented (9.9%) areas (χ2 = 37.49, *p* < 0.001). Higher educational status of mothers (AOR = 2.588, 95% CI: 1.220, 5.491), having open discussion with family (AOR = 3.175, 95%CI: 1.624, 6.206), having good knowledge (AOR = 4.511, 95% CI: 2.458, 8.278) and having positive attitude (AOR = 5.084, 95% CI: 2.764, 9.352) were factors positively associated with SRH services utilization.

**Conclusion:**

Compared with high schools from YFS implemented areas, the SRH service utilization was significantly lower among students from high schools where health facilities did not implement YFS. There is a need for enhancing efforts to establish YFS corners by the stakeholders at different hierarchies at places where the centers were not established so that SRH service uptake would be improved. In addition, it is better to promote open discussion with adolescents at the family level, and emphasis should be given for women education in the broad sense. Furthermore, wide-range awareness creation strategies should be used to address poor knowledge and negative attitude.

**Supplementary Information:**

The online version contains supplementary material available at 10.1186/s13690-020-00508-w.

## Background

The World Health Organization defines adolescence as the age group of 10–19 years old [1]. Adolescence is characterized by significant physiological, psychological, and social changes that put them for high risk of SRH problems [2, 3].

Young people from Sub-Saharan Africa countries are more at risk of SRH problems than those from the other parts of the world [4]. It is the hardest hit region in the world by the human immune-deficiency virus (HIV) with an estimated 22.5 million and 1.7 million people living with and having new infections respectively. Likewise, premarital sexual activity has the highest rate in sub-Saharan Africa; where more than half of girls aged 15–19 have sexual experience [5].

The concern for adolescent SRH services has been growing. This is mainly because they are vulnerable to health risks, especially those related to SRH [6, 7]. The most common adolescent problems related to sexuality and reproductive health include HIV/AIDS, unwanted pregnancy, unsafe abortion, early marriage, teenage pregnancy, and sexually transmitted infections (STIs) [7]. In Ethiopia, the SRH of young people has become a major public health concern. Gender inequality, sexual coercion, early marriage, polygamy, female genital mutilation, unplanned pregnancies, closely spaced pregnancies, STIs, and HIV/AIDS are among the many SRH problems faced by adolescents and youth in the country [8, 9]. Secondary school students are the most vulnerable group for SRH problems due to their inclination to be engaged in risky sexual behavior [10].

Despite the huge burden of SRH problems, adolescent SRH service is also not yet sufficiently organized by qualified and dedicated staff, space/room and time. Majority of health facilities are also providing sexual and reproductive health services as part of the routine health care with no particular focus to the services for adolescents and youths [11].

A good SRH is said to be present when one is able to have a safe and satisfying sex life, and is in a position to decide freely upon whether or not he/she wants to reproduce, and at what time in one’s life [12]. YFS is a wide-ranging SRH services which particularly address specific needs, desires and vulnerabilities of youths, prosper for attraction and retention of youths in a continuum of care [13].

To address the challenges faced by the youth, in 2009, the international non-governmental organizations (NGOs) such as the USAID flagship family planning and maternal, newborn, and child health program, the Integrated Family Health Program (IFHP) and other local NGOs began to expand YFS in six regions of Ethiopia in collaboration with the ministry of health. The Southern Nations Nationalities and Peoples Region (SNNPR) was one of the six. In line with the country’s policies and strategies, the YFS centers were built in university clinics, public health centers, and hospitals in the six regions of Ethiopia based on the recommendation from the regional health bureau and district health offices for the initial selection of the specific centers. During the selection, consideration of the vulnerability factors of the youth to various SRH problems, size of youth population in the area and existence of youth centers in the area for referral purpose were considered. The essence of the expansion was to create an environment that is tailored to age, sociocultural and economic contexts of youth and also to raise their awareness on SRH issues [11].

The services in the YFS range from provision of accurate and tailored information on a variety of SRH issues to provision of specific SRH services based on the needs of the youths. The SRH services are provided in the already existing health facilities by specially trained health care providers together with peer educators in a separate corner having a waiting and consultation spaces that are equipped with audiovisual materials and indoor games which put youths at ease while they visit the YFS sites. The service packages include: counseling and testing for HIV, other STIs and pregnancy; counseling for contraceptives, nutrition, and victims for sexual abuse and violence; offering full range of contraceptives, antenatal, post-natal and post abortion care; referral service for antiretroviral therapy, delivery and prevention of mother-to-child transmission of HIV, and promotion of condom. Youngsters access to YFS services in a convenient location and free of cost through the support of the peer educators who receive a 5 days training to assist youth clients by taking their cards from the triage to consultation room, provide health education to the youths and facilitate other activities in the YFS [14–16]. Since YFS centers have been established in the country for about a decade period, studies focused on the utilization, perspectives and quality of SRH services in the YFS corners in the country [13, 17–20].

To date, to the best of our knowledge, no study addressed the comparative disparities in sexual and reproductive health service utilization in YFS implemented and non-implemented areas of the country in general and our study area in particular. Therefore, this study compares SRH service utilization among youths in the high schools (grades 9–12) of YFS implemented and non-implemented areas in one of the peripheral districts in Southern Ethiopia, Ari district of South Omo Zone, to check whether a significant difference was there in the SRH service utilization and identify the factors which affect the SRH service use so that insights for intervention can be found for policy makers or programme planners.

## Methods and materials

### Study design

A school-based comparative cross-sectional study was conducted to compare the extent of SRH services utilization among students from YFS implemented and non-implemented areas and to identify the associated factors among adolescents from 1 to 30, March 2019.

### Study setting

South Ari is one of the districts in South Omo Zone and it is located 735 Km away from Addis Ababa, the capital city of Ethiopia. There were a total of six high schools in the district; two of them were located in YFS implemented areas of the district.

### Participants

The source population of this study was all adolescents between 15 and 19 years old who were attending their studies in the public high schools in South Ari district in 2019. The study population was all adolescents of age between 15 and 19 years in selected public schools during data collection period. Multi-stage cluster sampling technique was employed in order to select a representative sample of students. Four secondary schools (two from each of the YFS and non-YFS sites) were selected randomly out of the six secondary schools in the district. Samples were selected from the randomly selected schools based on proportion to the respective number of students. The total sample was allocated to each grade (9–12) in proportion to their student size. From each grade, sections were selected randomly. Finally, the study participants were selected by using computer-generated random numbers based on a sampling frame prepared by using their attendance sheet obtained from their respective schools. On the date of data collection, the randomly selected students were invited to the classes arranged in advance for data collection.

### Variables

The dependent variable of this study was SRH services utilization. Independent variables were predisposing, enabling and need factors of health care utilization. The predisposing factors considered in the study were sex, age, and place of residence and marital status (or relationship condition), knowledge and attitude. The enabling factors were access to services, cost of services, discussion of SRH issues with family members and perceived parental income status. The need factors include perceived need for SRH service, perceived personal health status, and worries (concern) about one’s health.

### Measurement

#### Data collection tools and procedures

The data collection instrument was a self-administered questionnaire developed after reviewing previous publications from similar or related literatures**.** The questionnaire was prepared in English and translated to Amharic and back to English to check for consistency of meaning. Data were collected by four diploma holding health workers and two bachelor holding supervisors from the health discipline respectively.

#### Operational definitions

Adolescent: In this study adolescent stands for boys and girls between the ages of 15–19 years.

Utilization of sexual and reproductive health services: This was measured through the dichotomous response (yes or no) by asking whether a participant had utilized one or more of SRH service components within the last 12 months. The positive response was further validated with questions on the type of SRH services utilized. A positive (“yes”) response to any one of these services was regarded as service utilization [21].

Knowledge of SRH services: Adolescents who scored above the mean of questions assessing participants’ knowledge were labeled as having good knowledge and those scored a mean or below the mean were considered as having poor knowledge [1].

Attitude towards SRH services: Attitude was measured by 7 items each having three categories of responses: disagree, neutral and agree. It was further recoded in to agree and disagree by considering being neutral to attitude questions as a disagreement. Finally, adolescents who scored above the mean for attitude questions were regarded as having a “positive attitude” and those who scored a mean or below the mean on the questions assessing attitude were regarded as having a “negative Attitude” [21].

#### Bias

Since the study was institution-based, there is a risk of contamination across observation units. Therefore, to minimize this, data were collected at the same time from the selected high schools. Since students were asked to remind sexual and reproductive health services provided to them in the last 1 year, this might lead to a recall bias.

To assure the quality of data, a day-long training was provided for data collection facilitators andsupervisors to brief them on the aims of the study, its significance and the contents of the instrument. The overall activity of data collection was closely supervised and coordinated by the principal investigator. The collected data were reviewed and checked for completeness and consistency onsite in the schools by the supervisors and data collection facilitators and before data entry by the research team. Pre-test was conducted in a high school at a nearby district on 5% of the sample size 2 weeks before commencement of the actual data collection and where appropriate, modification to wording and content of questions was made based on the results from the pretest. To reduce Hawthorne effect owing to the physical presence of the data collection facilitators in the class rooms, they were trained to remain at the stages of each class room with minimal movement in between the students’ seats.

### Sample size determination

The sample size for this study was determined based on assumptions of a 50% prevalence of SRH service utilization (as there was no study on the same topic) among school adolescents in the high schools where YFS was implemented (P2 = percent of exposed having the outcome = 50%) and a difference of 20% between YFS implemented and non-implemented areas (P1 = percent of unexposed having the outcome = 30%), a 5% level of significance and 80% power and a ratio of unexposed to exposed equal to 1. With these considerations, a sample size of 208 was calculated. A design effect of two was considered as the sampling procedure had involved a multistage cluster sampling and an additional 10% was considered to compensate for possible non-response. Finally, a total sample of 458 was calculated as a sample size for the study.

Based on findings from the study, power was calculated using STATA software Version 13 with a significance level of 0.05, sample size (N) = 458, P1 = proportion of youths from YFS not implemented areas and who utilized SRH services = 9.9% = 0.099, P2 = proportion of youths from YFS implemented areas and who utilized SRH services = 33.8% = 0.338, and allocation ratio = 1. Based on this assumptions and values, the power was estimated to be 1. Therefore, the sample calculated using this assumption was found to be adequate to show a significant difference in SRH utilization among the YFS implemented and non-implemented areas.

### Statistical methods

Data were entered in to Epi data version 4.4.1 software and then exported to SPSS version 20 for analysis. Descriptive statistics were computed and findings were summarized in tables and graphs with frequencies, mean, or standard deviations where appropriate. χ^2^ test was used to see significant difference in SRH service utilization among adolescents from YFS implemented and non-implanted areas. The association between SRH service utilization and the independent variables was examined by binary logistic regression. Crude odds ratios were computed to determine the strength of association of the selected explanatory variables with the dependent variable in the initial bivariable logistic regression analysis. To control for potential confounders and identify independent factors of SRH service utilization, variables which showed an association at a *p*-value ≤0.25 in the bivariable logistic regression model were selected as a potential candidate to fit to the final multivariable logistic regression analysis model. Model fitness was checked using Hosmer and Lemeshow goodness of fitness test (*p*-value ≥0.05). The association between SRH services utilization and the explanatory variables was reported by using odds ratio with its 95% CI and variables having *p*-value less than or equal to 0.05 in the multivariable logistic regression model were considered as statistically significant.

## Results

### Socio-demographic characteristics

A total of 426 participants (213 from YFS-implemented and 213 from non-implemented areas) were participated in the study yielding a response rate of 93.0%. The mean age of the respondents was 16.48 (SD ± 1.23). More than one-third of the study participants were grade nine students. About 58% of adolescents living in YFS implemented areas and 49% of adolescents living in YFS non- implemented areas were residing in rural areas and majority of them were Ari by ethnicity. Regarding the educational background of the adolescents’ mothers, 190 (44.6%) of them were having no formal education in both YFS-implemented and non-implemented areas (Table 1).

**Table 1:** Socio-demographic characteristics of study participants in YFS-implemented and non-implemented areas in South Ari district, March 2019 (*n* = 426)

Variables	Category	YFS-implemented (*n* = 213)	YFS non- implemented (*n* = 213)	Total (*n* = 426)
Sex	Male	136 (63.8)	133 (62.4)	269 (63.1)
	Female	77 (36.2)	80 (37.6)	157 (73.7)
Age	15–17	166 (77.9)	173 (81.2)	339 (79.6)
	18–19	47 (22.1)	40 (18.8)	87 (20.4)
Marital status	Single	143 (67.1)	168 (78.9)	311 (73.0)
	Married	18 (8.5)	13 (6.1)	31 (7.3)
	Has boy/girlfriend	45 (21.1)	30 (14.1)	75 (17.6)
	Others ^a^	45 (21.1)	2 (0.9)	9 (2.1)
Grade of education	Grade 9	69 (45.7%)	82 (54.3%)	151 (35.4%)
	Grade 10	70 (49.6%)	71 (50.4%)	141 (33.1%)
	Grade 11	41 (56.2)%	32 (43.8%)	73 (17.1%)
	Grade 12	33 (54.1%	28 (45.9%)	61 (14.3%)
Religion	Orthodox	52 (24.4)	67 (31.5)	119 (27.9)
	Protestant	144 (76.6)	127 (59.6)	271 (63.6)
	Muslim	15 (7.0)	15 (7.0)	30 (7.0)
	Others ^b^	2 (0.9)	4 (1.9)	6 (1.4)
Place of residence	Rural	123 (57.7)	104 (48.8)	227 (53.2)
	Urban	90 (42.3)	109 (51.2)	199 (46.7)
Ethnicity	Ari	146 (68.5)	132 (62)	278 (65.3)
	Amhara	28 (13.1)	33 (15.5)	61 (14.3)
	Goffa	18 (8.5)	24 (11.3)	42 (9.9)
	Malle	5 (2.3)	9 (4.2)	14 (3.3)
	Others^c^	16 (7.5)	15 (7)	31
Living arrangement	Living with single parents	51 (23.9)	70 (32.9)	121 (28.4)
	Living with both parents	88 (41.3)	78 (36.6)	166 (38.9)
	Living alone	4 (1.9)	7 (3.3)	11 (2.6)
	Living with boy/girlfriend	5 (2.3)	3 (1.4)	8 (1.9)
	Living with husband/wife	15 (7.0)	13 (6.1)	28 (6.6)
	Living with brothers/sisters	23 (10.8)	21 (9.9)	44 (10.3)
	Living with grandparents	27 (12.7)	21 (9.9%)	48 (11.3)
Father’s Educational Status	No formal education	65 (48.9%)	68 (51.1%)	133 (31.2%)
	Primary education	81 (44.0%)	103 (56.0%)	184 (43.2%)
	Secondary education and above	67 (61.5%)	42 (38.5%)	109 (25.6%)
Mother’s educational status	No formal education	94 (49.5%)	96 (50.5%)	190 (44.6%)
	Primary education	66 (45.8%)	78 (54.2%)	144 (33.8%)
	Secondary education	53 (57.6%)	39 (42.4%)	92 (21.6%)

Others ^a^: separated, divorced; others ^b^: Catholic, Adventist; Others ^c^: Wolayta, Oromo, Konso

### Sexual and reproductive health services utilization

**Fig. 1:**
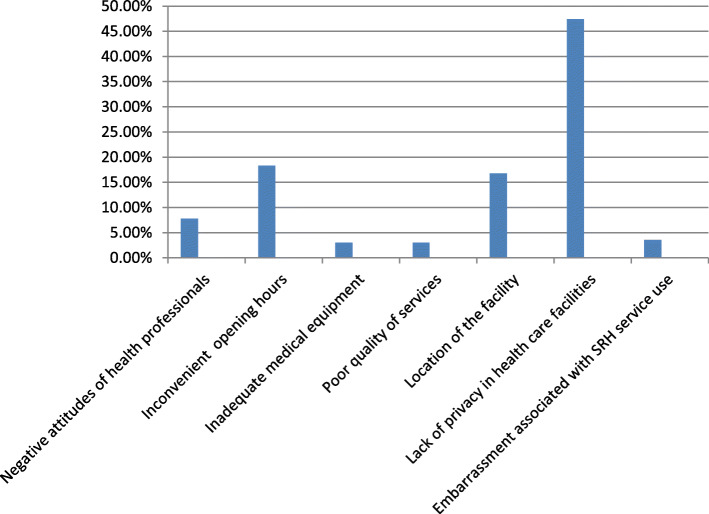
Students’ reasons for not using SRH services in South Ari district, South Omo zone, March 2019

Sexual and reproductive health services utilization in YFS-implemented and non-implemented areas were 33.8% [95% CI (28.2–40.4)] and 9.9% [95% CI (6.0–14.0)] respectively. The proportion of adolescents who used SRH services has significant difference between YFS-implemented and non-implemented areas (χ2 = 37.49, *p*-value < 0.001). The most common service utilized by adolescents was receiving information, education and communication materials (80.6%) followed by condom (57.0%) and voluntary counseling and testing for HIV (33.3%). The reasons mentioned by the participants for not using the SRH services were lack of privacy in health facilities, unfavorable health professionals’ attitude, embarrassment and perceived inadequate medical equipment (Fig. 1).

### Knowledge and attitude of participants about SRH services

Out of the study participants, 53.35% were not aware of at least one of the SRH services provided in the YFS centers. About two-third (64.6%) of the adolescents had poor knowledge about SRH services (Table 2).

**Table 2:** Knowledge of the study participants related to SRH service in YFS-implemented and non-implemented areas in South Ari district, March 2019 (*n* = 426)

Variables Respondent’ knew about:	Yes Frequency (%)	No Frequency (%)
At least one SRH service	199 (46.7%)	227 (53.3%)
Information, education and communication related to SRH issues	162 (38.0%)	264 (62.0%)
Family Planning services	181 (42.5%)	245 (57.5%)
Pregnancy testing and care	26 (6.1%)	400 (93.9%)
Treatment of Sexually transmitted Infections	21 (4.9%)	405 (95.1%)
VCT for HIV	150 (35.2%)	276 (64.8%)
Condom provision service	164 (38.5%)	262 (61.5%)
Cervical cancer screening	36 (8.5%)	390 (01.5%)
Teenage pregnancy can lead to maternal and infant death	240 (56.3%)	186 (43.7%)
Unwanted pregnancy can be prevented by using condoms	334 (78.4%)	92 (21.6%)
Reproductive tract infections can be caused by unprotected sex	63 (14.8%)	363 (85.2%)
Sexually transmitted diseases can be acquired by unprotected sexual intercourse	359 (84.3%)	67 (15.7%)
HIV/AIDS can be prevented by avoiding unprotected sexual contact.	347 (81.5%)	79 (18.5%)

Among 426 participants, 83.3% agreed that using condom is a sign of not trusting one’s partner. Eleven percent of the participants agreed that a boy/girl should have sex before he/she gets married and 31.2% of them agreed that discussing on condom or other contraceptive methods with young people promotes promiscuity. Sixty one percent of them believed that the risk of acquiring HIVAIDS infection can be reduced by receiving VCT services. Regarding their preference to visit health care facilities, 52 % preferred to visit health facilities to get SRH services. By their preference to age and sex of health care service providers, 53.0 and 49.1% agreed that they do not have special preference to age and sex of the health care providers involved in SRH service provision. Nearly 19.0% of adolescents reported that their culture prohibited them from utilization of SRH services. With consideration of the mean score of all attitude questions, 295(69.2%) of the study participants had positive attitude towards SRH services whereas the rest had negative attitude.

### Comparison of participants’ knowledge and attitude by YFS implementation status

Regarding participants’ attitude towards SRH services; more than a third and about a quarter of adolescents (35.7 and 25.8%) from YFS implemented and non-implemented areas respectively (χ^2^test *p*-value = 0.027). Likewise, 41.3 and 29.6% of participants with good knowledge were from YFS implemented and YFS non-implemented areas respectively (χ^2^ test p-value = 0.01).

### The enabling factors of participants

Among adolescents participated in this study, 20.0 and 59.4% of them had experienced open discussion with their families and with their peers on matters related to SRH issues respectively. From the total participants, 33.3, 59.4 and 7.3% of them perceived their family economic status as poor, average and rich respectively.

### The need factors of participants

Out of the respondents, 26.8, 54.2, 17.4 and 1.6% perceived their health condition as very good, good, fair and ill respectively. Concerning worry about their own current health status (previous 12 months), 12, 79.3, and 8.7% were not concerned, having some degree of concern and extremely concerned respectively.

### Factors associated with utilization of SRH services

In the final multivariable binary logistic regression model, living in YFS implemented areas, mother’s educational status, discussion of SRH issues with their family, knowledge and attitude towards the SRH services remained significantly associated with utilization of SRH services.

The odds of utilizing SRH service among adolescents who were from YFS implemented areas were four times higher compared with their counterparts (AOR **=**4.076, 95%CI: 2.150, 7.727). Likewise, the odds of utilizing SRH services among adolescents whose mothers’ attended secondary education and above was 2.58 times higher than those with mothers having no formal education (AOR = 2.588, 95% CI: 1.220, 5.491). Compared with their counter parts, the odds of utilization of SRH services was also higher among adolescents who discussed SRH issue with their families (AOR = 3.175, 95%CI: 1.624, 6.206), who had good knowledge (AOR = 4.511, 95% CI: 2.458, 8.278), and positive attitude (AOR = 5.084, 95% CI: 2.764, 9.352) (Table 3).

**Table 3:** Multivariable logistic regression analysis to identify associated factor of SRH service utilization in South Ari district, March 2019 (*n* = 426)

Variables	Category	utilization of SRH services		COR [95% C.I]	AOR [95% C.I]	*P*-value
		Not utilized	Utilized			
YFS implementation status	YFS non- implemented	192 (90.1%)	21 (9.9%)	1	1	
	YFS implemented	141 (66.2%)	72 (33.8%)	4.66 [2.74,7.95]	**4.07 [6 2.150, 7.727]**	.001
Grade of education	Grade 9	125 (82.8%)	26 (17.2%)	1	1	
	Grade 10	101 (71.6%)	40 (28.4%)	1.94 [1.089,3.33]	2.21 [1.070,4.591]	0.032
	Grade 11	55 (75.3%)	18 (24.7%)	1.57 [0.79,3.10]	1.80[.737,4.428]	0.196
	Grade 12	52 (85.2%)	9 (14.8%)	0.83 [0.365,1.89]	.92 [.329, 2.565]	0.870
Mother’s educational status	No formal education	166 (87.4%)	24 (12.6%)	1	1	0.028
	Primary education	109 (75.7%)	35 (24.3%)	2.22 [1.25,3.93]	2.069 [1.021,4.191]	0.044
	secondary education and above	58 (63.0%)	34 (37.0%)	4.05 [2.22,7.40]	**2.588 [1.220, 5.491]**	0.013
Discussion on SRH issue with family members	Yes	42 (48.8%)	44 (51.2%)	6.22 [3.69,10.46]	**3.17 [1.624, 6.206]**	0.001
	No	291 (85.6%)	49 (14.4%)	1	1	
Perceived health status	Very good	84 (73.7%)	30 (26.3%	1	1	0.093
	Good	177 (76.6%	54 (23.4%)	2.14 [0.24,18.53]	1.22 [.102, 14.643]	0.874
	poor	66 (89.2%)	8 (10.8%)	1.83 [0.21,15.54]	1.18 [.102, 13.585]	0.895
	Ill health	6 (85.7%)	1 (14.3%	0.72 [0.77,6.83]	.36 [.028, 4.640]	0.436
knowledge	Good	87 (57.6%)	64 (42.4%)	6.24 [3.77,10.31]	**4.51 [2.458, 8.278]**	.001
	Poor	246 (89.5%)	29 (10.5%)	1	1	
Attitude	Positive	73 (55.7%)	58 (44.3%)	5.90 [3.60,9.66]	**5.08 [2.764, 9.352]**	.001
	Negative	260 (88.1%)	35 (11.9%)	1	1	

## Discussion

This study aimed to compare differences in SRH services utilization among high school adolescents from youth friendly service implemented and non-implemented areas, and to identify independent factors for SRH service utilization.

In the study, adolescents from high schools where health facilities had not yet implemented YFS had a lower utilization of SRH services compared with health facilities which implemented YFS. This can be justified as having access to various SRH services in an environment that is tailored to adolescent’s age, sociocultural and economic contexts would increase the utilization of the services [11]. Furthermore, adolescents living in YFS implemented areas have higher probability of getting information regarding SRH services and health professionals working in the YFS centers may have brought change in norms of the community living around the centers [22]. The finding highlights a need for scale up of the existing YFS sites to non-implemented sites so that SRH services can be promoted to all adolescents and youths.

Majority of the respondents in the present study had not utilized SRH services owing to varied reasons. Lack of privacy was the most commonly reported reason for not using SRH services. Health professionals’ attitude, embarrassment in receiving the services and perceived inadequacy of medical equipment were also the reasons listed by the participants. This finding was consistent with findings from studies conducted in East Gojam, Nekemte, and Tanzania [6, 21, 23]. Mostly, they feared that their parents would find out their visit to the clinic. This implies that there is a need of tackling the barriers by dealing with health professionals, community leaders and with the adolescents themselves. Due emphasis should be given to increase the capacity of health care providers so that they can deliver services without imposing their own and socially endorsed moral frameworks on the adolescents’ sexual behavior. In addition, there is a need to address the cultural, religious and traditional value systems that prevent health professionals from providing a better quality and comprehensive SRH services to the adolescents. The current study showed that adolescents’ whose mother’s attended secondary and higher education were having higher odds of utilization of SRH services. This finding is in agreement with a study conducted in Asgede-Tsimbla district, East Gojam and Gondar [1, 6, 24]. This can be explained that women at higher level of education might have been more open to discuss SRH issues with their children. It might have also been related to the fact that women at higher level of education would have a better access to SRH messages and would be more flexible to deal with their children or investigate for any problems their children encounter regarding SRH services use. In contrary, studies from Debre Berhan and Woreta towns showed no association between maternal education and adolescents’ SRH service utilization [5, 10]. The variation might have been emanated from socio-cultural differences in the Northern and Southern Ethiopian regions. In the current study, knowledge and attitude of adolescents on SRH services showed a significant relationship with the presence or absence of YFS centers in their locality. Positive attitude and good knowledge was higher among adolescents from schools where YFS centers were established in the health centers. It is plausible that adolescents living in areas where youth friendly services were established would get appropriate information from their in-school and out of school peers through the organized peer education delivered in the YFS centers. The presence of the peer support system could be a source of attraction to the youngsters and might have resulted in having a good knowledge and favorable attitude since trained health care providers and the peers provide health education to youths in the YFS implemented areas [14–16].

Likewise, the odds of utilizing SRH services were higher among adolescents’ having good knowledge than their counterparts. This finding is consistent with the studies conducted in East Gojam, Harar town, Lao PDR and North Shewa zone [6, 13, 25, 26]. This can be justified as adolescents with good knowledge had adequate information regarding the consequences of SRH problems. It is better to use YFS sites as a learning center for adolescents as lack of knowledge makes them vulnerable to unsafe reproductive health behavior and inappropriate choices. Some of these choices may have undesirable effects on their reproductive health in the future such as unplanned pregnancy, STI infection, HIV/AIDS and other sexual and reproductive health problems. The effects of these wrong choices are manifold with some capable of lasting for a lifetime. These potential human resource and future leaders end up as school dropouts. Additionally, these would have social and economic implications to their households and the nation as a whole.

The current study assured that adolescents with favorable attitude towards SRH services were more likely to utilize SRH service as compared to their counterparts. This could be explained by the fact that having a positive feeling towards the services derives initiation to seek SRH services. The finding is supported by the study conducted in Lao PDR [25] which reported that the prevailing negative cultural attitudes were the main barrier for Lao youths to access SRH services.

Moreover, this study showed that adolescents who ever discussed on SRH issues with their families were more likely to utilize SRH services than adolescents who never discussed on SRH issues with anyone. This finding is analogues with other studies elsewhere, which reported ever discussion on SRH issues was an independent predictor for SRH service utilization among adolescents [1, 5, 17]. Communicating SRH issues with parents is very crucial for adolescents so as to advance their awareness of SRH issues. Moreover, such discussions (especially if the family members have good knowledge on reproductive health problems and reproductive health services) increase adolescents’ feeling of self-trust and there by urge their SRH seeking behavior. This implies that adolescents who discuss SRH issues with their family would have a better knowledge and awareness about SRH services and thus would be motivated to use the services [27]. In contrary, finding of this study is not supported by the study conducted in North Shewa which reported that adolescents who never discussed on VCT services were significantly more likely to use the service than adolescents who had discussed on the service [26]. This difference can be justified by adolescents who have information and have discussed SRH services with different individuals may not think they need SRH service because they perceive a low risk, which shows there is a gap in continuity of discussion, communication, and information to bring behavioral change. These contradictory findings call for a further investigation to the effect of discussion on SRH issues with a family or peers on the subsequent SRH service uptake.

### Policy recommendations

There is a need for more efforts in order to scale up SRH services in YFS non implemented areas among the stakeholders at different hierarchies. In addition, it is better to promote open discussion with adolescents at the family level, and emphasis should be given for women education in the broad sense. Furthermore, wide-range awareness creation strategies should be used to address poor knowledge and negative attitude.

### Strength and limitations of the study

This study is the first of its kind in comparing SRH service utilization among adolescents in the YFS implemented and non-implemented areas. Moreover, the power estimated after conducting the study was high which refers to the inclusion of adequate samples to see the difference in the two groups.

However, the study has some limitations to consider. As this study was cross-sectional, the factors do not establish temporal relationship; therefore, inference of causation is not possible. Had it not been for resource limitations, the study would have been better if it were a longitudinal study which compares SRH service among adolescents in YFS implemented and non-implemented areas. However, this cross-sectional comparison would provide useful information as an input to future studies of longitudinal nature. Recall bias may also affect responses related to SRH service use over the last 1 year and this might have resulted in either over or under reporting for the presence of both undesirable and desirable sexual and reproductive health practices and events as responses in the questions [28]. The recall bias might be more of a concern among YFS-non implemented areas as these areas had a lesser chance of getting peer education which would help as a cue for memory. There could also be a social desirability bias particularly among adolescents from YFS implemented areas for such adolescents might expect that SRH service use is appropriate to their family and society at large. Moreover, the quantitative study did not allow for probing into certain variables like cultural issues and perception. This implies a need for further qualitative study to complement the findings of this study.

## Conclusion and recommendations

There was a significant difference in SRH service utilization among adolescents between YFS implemented and YFS non- implemented areas. The utilization of SRH services was low among students from high schools where health facilities had not yet implemented YFS compared with health facilities which implemented YFS. Higher level of mothers’ educational status, open discussion with family, having good knowledge and positive attitude were significant factors associated with SRH service utilization.

## Supplementary Information

**Additional file 1.**

## Data Availability

The datasets used and/or analyzed during the current study available from the corresponding author on reasonable request.

## Abbreviations

AIDS: Acquired Immune Deficiency Syndrome
SRH: Sexual and reproductive health
YFS: Youth-Friendly Services

## References

[1] Gebreyesus H, Teweldemedhin M, Mamo A (2019). Determinants of reproductive health services utilization among rural female adolescents in Asgede-Tsimbla district northern Ethiopia: a community based cross-sectional study. Reprod Health.

[2] Introduction to adolescence and to adolescent health. Paper presented at: Training Course in Sexual and Reproductive Health Research. Geneva: 2012. Available from: https://www.gfmer.ch/SRH-Course-2012/adolescent-health/Introduction-adolescence-adolescent-health-WHO-2012.htm.

[3] Dehne KL, Riedner G, Berer M, Organization WH (2005). Sexually transmitted infections among adolescents: the need for adequate health services.

[4] Ringheim KG (2010). Improving the Reproductive Health of sub-Saharan African Youths:Arouteto achieve the Millenium Development Goals.

[5] Tlaye KG, Belete MA, Demelew TM (2018). Reproductive health services utilization and its associated factors among adolescents in Debre Berhan town, Central Ethiopia: a community-based cross-sectional study. Reprod Health.

[6] Abajobir AA, Seme A (2014). Reproductive health knowledge and services utilization among rural adolescents in east Gojjam zone, Ethiopia: a community-based cross-sectional study. BMC Health Serv Res.

[7] Helamo D, Kusheta S, Bancha B, Habtu Y, Yohannes S (2017). Utilization and factors affecting adolescents and youth friendly reproductive health services among secondary school students in Hadiya zone, southern nations, nationalities and peoples region, Ethiopia. Int J Pub Health Safe.

[8] Tegegn A, Gelaw Y. Adolescent reproductive health services in jimma city: accessibility and utilization. Ethiopian J Health Sci. 2009;19(2):91–102. 10.4314/ejhs.v19i2.69414.

[9] Feleke SA, Koye DN, FelekeDemssie A, Mengesha ZB (2012). Reproductive health service utilization and associated factors among adolescents (15–19 years old) in Gondar town, Northwest Ethiopia. BMC Health Serv Res.

[10] Abate AT, Ayisa AA, W/Mariam TG (2019). Reproductive health services utilization and its associated factors among secondary school youths in Woreta town, South Gondar, North West Ethiopia: a cross sectional study. BMC Res Notes.

[11] USAID, Pathfinder and JSI. Integrated family health programme. Integrating Youth-Friendly Services into Public Health Facilities; National adolescent and youth strategy (2006–2015). http://www2.pathfinder.org/site/DocServer/Integrating_YouthFriendly_Services_into_Public_Health_F.pdf?docID=20062.

[12] Health Poverty action. https://www.healthpovertyaction.org/how-poverty-is-created/women-girls/sexual-reproductive-health/..

[13] Motuma (2016). Utilization of youth friendly services and associated factors among youth in Harar town, east Ethiopia: a mixed method study. BMC Health Serv Res.

[14] Scholl E, Schueller J, Gashaw M, Wagaw A, Wolde ML. Assessment of youth reproductive health programs in Ethiopia: Youth Net Assessment Team; 2004. Available at: https://www.fhi360.org/sites/default/files/media/documents/AssessmentofYouthReproductiveHealthProgramsinEthiopia.pdf.

[15] National Adolescent and Youth Reproductive Health Strategy, 2006–2015. Addis Ababa: Federal Democratic Republic of Ethiopia Ministry of Health; 2006.

[16] National Adolescent and Youth Reproductive Health Strategy, 2016-2020. Addis Ababa: federal democratic republic of Ethiopia ministry of health, 2016.

[17] Ayehu A, Kassaw T, Hailu G (2016). Level of young people sexual and reproductive health service utilization and its associated factors among young people in Awabel district, northwest Ethiopia. PLoS ONE.

[18] Mauerhofer A, Berchtold A, Michaud PA, Suris JC (2010). Female adolescents’ views on a youth-friendly clinic. Swiss MedicalWeekly.

[19] Mulugeta (2019). Assessment of Youth-Friendly Service Quality and Associated Factors at Public Health Facilities in Southern Ethiopia: A Facility-Based Cross-Sectional Study. BioMed Res Int.

[20] Kahsay K, Berhe S, Alemayehu M (2016). Utilization of youth friendly services and associated factors in Mekelle town, Tigray, Northern Ethiopia. Int J Ther Appl.

[21] Binu W, Marama T, Gerbaba M, Sinaga M (2018). Sexual and reproductive health services utilization and associated factors among secondary school students in Nekemte town, Ethiopia. Reprod Health.

[22] Jain A, Ismail H, Tobey E, Erulkar A (2017). “Understanding Adolescent and Youth Sexual and Reproductive Health-seeking Behaviors in Ethiopia: Implications for Youth Friendly Service Programming,” Research Report.

[23] Mbeba RM (2012). Barriers to sexual reproductive health services and rights among young people in Mtwara district, Tanzania : a qualitative study. Pan Afr Med J.

[24] Feleke SA, Koye DN, Demssie AF, Mengesha ZB (2013). Reproductive health service utilization and associated factors among adolescents (15–19 years old) in Gondar town, Northwest Ethiopia. BMC Health Serv Res.

[25] Thongmixay S, Essink DR, Greeuw TD, Vongxay V, Sychareun V, JEW B (2019). Perceived barriers in accessing sexual and reproductive health services for youth in Lao People’s Democratic Republic. PLoS ONE.

[26] Negash W (2016). Reproductive health service utilization and associated factors: the case of north Shewa zone youth, Amhara region, Ethiopia. Pan Afr Med J.

[27] Abraham G, Yitbarek K, Morankar SN (2019). Determinants of adolescents reproductive health service utilization in Ethiopia: a systematic review of quantitative evidence. Adolesc Health Med Ther.

[28] Spencer EA, Brassey J, Mahtani K, Catalogue of Bias Collaboration (2017). Recall bias. Catalogue Of Bias.

