# Supplementary material for: Disparities in utilization of sexual and reproductive health services among high school adolescents from youth friendly service implemented and non-implemented areas of Southern Ethiopia
Source: Arch Public Health. 2020 Dec 1;78:126. doi: 10.1186/s13690-020-00508-w (PMC7709284; doi:10.1186/s13690-020-00508-w)
Supplement: Supplementary file 1 — Additional file 1. [file 13690_2020_508_MOESM1_ESM.docx]

| Questionnaire code: | | | | | | | | | |
| --- | --- | --- | --- | --- | --- | --- | --- | --- | --- |
| **PART ONE: SOCIO-DEMOGRAPHIC CHARACTERISTICS** | | | | | | | | | |
| S.N | Questions | Response | | | | | |  | |
| 101. | Sex of the respondent | 1. Male 2. Female | | | | | |  | |
| 102. | Age respondent | ___________________ | | | | | |  | |
| 103. | What is your relationship status? | 1. Single  2. Married  3. Have a boy/girl friend  4. Other___________. | | | | | |  | |
| 104 | What is your academic class? | 1. Grade 9 2. Grade10 3. Grade 11 4. Grade 12 | | | | | |  | |
| 105 | What is your religion | 1. Orthodox 2. Protestant 3. Muslim 4. Other,specify | | | | | |  | |
| 106 | Where did you live? | 1.Rural 2. Urban | | | | | |  | |
| 107 | What is your ethnicity? | 1. Ari 2. Amhara 3. Male 4. Goffa 5. If other specify_____________ | | | | | |  | |
| 108 | What are your living arrangements? | 1. With Father or Mother only  2.With Father and Mother  3. Alone  4. With girlfriend/ boyfriend  5. living with husband/wife  6.living with grand parents  7. other, specify | | | | | |  | |
| 109 | What is the educational status of your father? | 1. No formal Education  2. Primary education  3. Secondary education and above | | | | | |  | |
| 110 | What is the educational status of your mother? | 1. No formal Education  2. Primary education  3. Secondary education and above | | | | | |  | |
| 111. | Is there any part of your culture that prohibits utilization of adolescent reproductive health? | 1.YES  2. NO. | | | | | |  | |
|  | **PART TWO: DISCUSSION OF SRH ISSUES.** | | | | | | | | |
| 201. | Have you ever discussed sexual and reproductive health issues with your parents? | 1.yes  2. No | | | | | |  | |
| 202. | Have you ever discussed SRH issues with your peers? | 1.Yes  2. No. | | | | | |  | |
|  | **PART THREE: KNOWLEDGE ON REPRODUCTIVE HEALTH SERVICES** | | | | | | | | |
| 301. | Do you know sexual and reproductive health services? | | | | 1.Yes | | 0.No. | If ‘No,’ skip to Q#311. | |
| 302. | If yes to Q_302_, Which one? | | | | | | |  | |
| 303. | Information in SRH issues | | | | 1.yes | | 0. No |  |  |
| 304. | Family Planning services | | | | 1.yes | | 0. No |  |  |
| 305. | Pregnancy testing and care | | | | 1.yes | | 0. No |  |  |
| 306. | Sexually transmitted Infections treatment | | | | 1.yes | | 0. No |  | |
| 307. | VCT for HIV | | | | 1.yes | | 0. No |  |  |
| 308. | Condom provision service | | | | 1.yes | | 0. No |  |  |
| 309. | Cervical cancer screening | | | | 1.yes | | 0. No |  |  |
| 311. | teenage pregnancy can lead to maternal and infant death? | | | | 1.Yes | | 0. No |  | |
| 312. | Unwanted pregnancy can be prevented by using condoms | | | | 1.YES | 0. No | |  | |
| 313. | Reproductive tract infections can be caused by unprotected sex | | | | 1.Yes | 0. No | |  | |
| 314. | Sexually transmitted diseases can be acquired by unprotected sexual intercourse | | | | 1.Yes | 0. No | |  | |
| 315. | HIV/AIDS can be prevented by avoiding unprotected sexual contact. | | | | 1. Yes | 0. No | |  | |
| **PART FOUR: ATTITUDE RELATED QUESTIONS** | | | | | | | | | |
| 401. | Using condom is a sign of not trusting your partner | 1. Agree 2. Not sure 3. Disagree | | | | | |  | |
| 402. | A boy/girl should have sex before he/she gets married | 1. Agree 2. Not sure 3. Dis agree | | | | | |  | |
| 403. | Discussing condom or other contraceptive methods with young people promotes  Promiscuity | 1. Agree 2. Not sure 3. Disagree | | | | | |  | |
| 404. | Believe that risk of AIDS can be reduced by VCT services | 1. Agree 2. Not sure 3. Disagree | | | | | |  | |
| 405. | I prefer to visit health facilities to get sexual and reproductive health services | 1. Agree 2. Not sure 3. Disagree | | | | | |  | |
| 406. | There is no problem if service provider of sexual and reproductive services is either young or adult. | 1. Agree 2. Not sure 3. Disagree | | | | | |  | |
| 407. | There is no problem if service provider of sexual and reproductive services can be male or female. | 1. Agree 2. Not sure 3. Disagree | | | | | |  | |
|  | **PART FIVE: ENABLING FACTORS** | | | | | | | | |
| 501. | is youth friendly service implemented in your living area? | 1.Yes  2.No. | | | | | |  | |
| 503. | How long does it take to reach the ARHS/SRH providing center | 1. 30hr  2. 30-1 hr.  3. 1hr -2 hrs.  4. > 2 hours | | | | | |  | |
| 503 | Economic status of the family | 1. Poor 2. Average 3. rich | | | | | |  | |
|  | **PART SIX: NEED FACTORS** | | | | | | | | |
| 601. | How do you perceive about your health status? | 1. Very good. 2. Good 3. Fair 4. Ill health | | | | | |  | |
| 602. | Have you been concerned or worried about your health status in the last 12 months? (**Tick one box only**) | 1. Not concerned at all. 2. Somewhat concerned. 3. Extremely concerned. | | | | | |  | |
| 603. | Generally, how do you explain about your need to get SRHS **(Tick one box only)**? | 1. I don’t need at all. 2. I need it slightly. 3. I need it seriously. | | | | | |  | |
|  | **PART SEVEN: QUESTIONS RELATED TO UTILIZATION OF SEXUAL & REPRODUCTIVE HEALTH SERVICES** | | | | | | | | |
|  | Ever utilized at least one of sexual and reproductive health services in the health facilities? | | 1.Yes  2. No. | | | | | | |
| 701. | Have you utilized one of sexual and reproductive health services in the last 12 months? | | | 1.Yes  2. No. | | | | | If No, go to 703 |
| 702. | If yes which SRH services did you utilize (more than one answer is possible)? | | | | | | | |  |
|  | Information in SRH issues | | | 1. Yes 0. No | | | | |  |
|  | VCT service for HIV | | | 1. Yes 0. No | | | | |  |
|  | Family Planning services | | | 1. Yes 0. No | | | | |  |
|  | STIs treatments | | | 1. Yes 0. No | | | | |  |
|  | Condom provision services | | | 1. Yes 0. No | | | | |  |
|  | Pregnancy testing and care | | | 1. Yes 0. No | | | | |  |
|  | Post abortion care | | | 1. Yes 0. No | | | | |  |
|  | Cervical cancer screening | | | 1. Yes 0. No | | | | |  |
| 703 | Reason not to utilize sexual and reproductive health services | | | 1. Negative attitude of health professionals 2. Inconvenience opening hour 3. Inadequate medical equipment 4. Poor quality of the services 5. Locations of the facility 6. Lack privacy in the health care facilities 7. Embarrassment associated with SRH service use 8. Other,specify | | | | |  |
